# Vitamin D supplementation to prevent depression and poor physical function in older adults: Study protocol of the D-Vitaal study, a randomized placebo-controlled clinical trial

**DOI:** 10.1186/s12877-015-0148-3

**Published:** 2015-11-19

**Authors:** Elisa J. de Koning, Natasja M. van Schoor, Brenda W.J.H. Penninx, Petra J.M. Elders, Annemieke C. Heijboer, Jan. H. Smit, Pierre M. Bet, Maurits W. van Tulder, Martin den Heijer, Harm W.J. van Marwijk, Paul Lips

**Affiliations:** EMGO Institute for Health and Care Research, VU University Medical Center, P.O. Box 7057, 1007 MB Amsterdam, The Netherlands; Department of Epidemiology and Biostatistics, VU University Medical Center, P.O. Box 7057, 1007 MB Amsterdam, The Netherlands; Department of Psychiatry, VU University Medical Center/GGZ inGeest, A.J. Ernststraat 1187, P.O. Box 1081 HL, Amsterdam, The Netherlands; Department of General Practice and Elderly Care Medicine, VU University Medical Center, P.O. Box 7057, 1007 MB Amsterdam, The Netherlands; Department of Clinical Chemistry, Endocrine Laboratory, VU University Medical Center, P.O. Box 7057, 1007 MB Amsterdam, The Netherlands; Department of Clinical Pharmacology and Pharmacy, VU University Medical Center, P.O. Box 7057, 1007 MB Amsterdam, The Netherlands; Department of Health Sciences, VU University, P.O. Box 7057, 1007 MB Amsterdam, The Netherlands; Department of Internal Medicine, Endocrine section, VU University Medical Center, P.O. Box 7057, 1007 MB Amsterdam, The Netherlands; Primary Care Research Centre, Institute of Population Health, University of Manchester, Oxford Road, Manchester, M13 9PL United Kingdom

**Keywords:** Vitamin D, Depressive symptoms, Physical functioning, Functional limitations, Physical performance, Older adults, Randomized clinical trial, Prevention, Supplementation

## Abstract

**Background:**

Depressive symptoms and decreased physical functioning are interrelated conditions and common in older persons, causing significant individual and societal burden. Evidence suggests that vitamin D supplementation may be beneficial for both mental and physical functioning. However, previous randomized controlled trials have yielded inconsistent results and often had suboptimal designs. This study examines the effect of vitamin D supplementation on both depressive symptoms and physical functioning in a high-risk population of older persons with low vitamin D status.

**Methods/design:**

The D-Vitaal study is a randomized, double-blind, placebo-controlled trial investigating the effects of a daily dose of 1200 IU vitamin D_3_ versus placebo for one year on depressive symptoms and physical functioning (primary outcomes) in older adults. Participants (*N* = 155, age 60–80 years) were recruited from the general population. Eligibility criteria included the presence of depressive symptoms, ≥1 functional limitation and serum 25-hydroxyvitamin D levels between 15 and 50/70 nmol/L (depending on season). Secondary outcomes include incidence of major depressive disorder, anxiety symptoms, health-related quality of life, cognitive function and cost-effectiveness of the intervention.

**Discussion:**

With this study, we aim to elucidate the effects of vitamin D supplementation on depressive symptoms and physical functioning in older persons who are at high risk of developing more substantial mental and physical problems. If effective, vitamin D supplementation can be a preventive intervention strategy that is easy to implement in the primary care setting.

**Trial registration:**

Netherlands Trial Register NTR3845. Registered 6 February 2013.

## Background

Depressive symptoms are common in older adults, occurring in 8-16 % of persons over 55 years of age [1]. These symptoms are associated with various adverse health outcomes, such as a higher risk of cardiovascular diseases [2], hospitalization and mortality [3] and reduced quality of life [1]. Treatment of depression in older persons is often suboptimal, for example due to societal stigma, side-effects of anti-depressant medication, or interactions of antidepressants with other medications [2, 4]. Hence, development of a simple and safe prevention strategy is pivotal.

Ageing is also commonly accompanied by a decline of physical functioning. Studies show that functional limitations and poor physical performance are highly interrelated with depressive symptoms, both cross-sectionally and longitudinally [2, 5–9], which can easily result in a downward spiral.

Previous research suggests that vitamin D supplementation may improve both mental and physical health, although evidence is inconsistent [10–12]. Vitamin D inadequacy - defined as serum 25-hydroxyvitamin D (25(OH)D) levels of <50 nmol/L [13] - occurs in about 50 % of elderly persons from Western countries [14]. Vitamin D is synthesized in the skin under the influence of sunlight. In addition, some vitamin D is retrieved from food, especially from fatty fish [15]. Causes of vitamin D deficiency in older persons include declining efficiency of the skin to synthesize vitamin D, a lower amount of sun exposure and reduced nutritional intake [16].

**Fig. 1:**
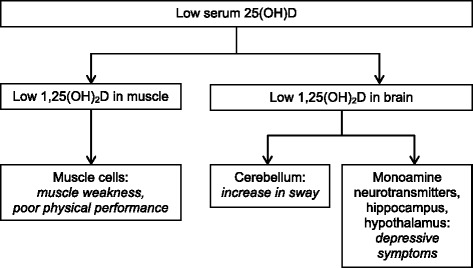
Pathophysiological effects of low vitamin D status on mental and physical functioning

Several biological mechanisms that can explain the relationship of vitamin D deficiency with depressive symptoms and poor physical functioning have been suggested (see also Fig. 1). The active metabolite of vitamin D - 1,25 dihydroxyvitamin D (1,25(OH)_2_D) - is synthesized in the brain by the enzyme 1α-hydroxylase [17], enabling local activation of vitamin D. Moreover, the vitamin D receptor (VDR) is present in several brain areas important for depression and emotional behaviour, including the hippocampus and hypothalamus [18]. Furthermore, 1,25(OH)_2_D promotes the synthesis of depression-related monoamine neurotransmitters such as serotonin [17, 19, 20] and has a general protective effect on brain functioning through immunomodulation, anti-inflammatory action and promotion of neuroplasticity [17, 19, 21]. Regarding physical functioning, the VDR has been observed in the cerebellum [18], which is an important brain area for mobility, gait and balance [22]. In addition, the presence of 1,25(OH)_2_D and the VDR in muscle tissue facilitates muscle contraction speed, muscle power and cell growth [19].

Prospective cohort studies suggest that vitamin D deficiency is associated with depressive symptoms, poor physical performance and functional limitations [23–26]. In the InChianti study, vitamin D deficiency at baseline almost doubled the risk of depressive symptoms after three and six years and was also associated with lower physical performance [27].

Evidence from randomized controlled trials (RCTs) is diffuse and inconclusive. Tables 1 and 2 provide an overview of previous RCTs examining the effects of vitamin D supplementation on depressive symptoms and physical functioning. Only RCTs that included adults and had a sample size of ≥40 were selected (search strategy available from the author). From these tables, it can be concluded that previous RCTs have been heterogeneous with respect to sample size, age range of the participants, dosage of the supplementation, duration of the intervention and outcome measures. Moreover, only a very limited amount of studies included high-risk participants, i.e. persons with low serum 25(OH)D and mental/physical symptoms at baseline. It can be expected that supplementation is more effective and relevant in persons with low baseline 25(OH)D levels and in persons with clinically relevant depressive symptoms or reduced physical functioning.

**Table 1:** Overview of RCTs that examined the effect of vitamin D supplementation on depression

Trial, year	Participants	Low vitD as inclusion criterion?	Depressive symptoms as inclusion criterion?	Intervention	Outcome	Results
Lansdowne et al., 1998 [68]	44 healthy adults, age 18–43 yrs., 77 % female.	No	No	400 or 800 IU/day vitD_3_ vs. placebo for 5 days in late winter.	Positive and negative affect (PANAS).	Improved positive affect, possibly reduced negative affect in both vitD groups.
Jorde et al., 2008 [69]	441 overweight/obese outpatients or community dwellers, age 21–70 yrs., 64 % female.	No	No	20,000 or 40,000 IU/week vitD_3_ vs. placebo for 1 yr.	Depressive symptoms (BDI).	Lower depression scores in both vitD groups. Effect more pronounced in persons with higher baseline depression scores.
Sanders et al., 2011 [70]	2012 community dwelling women, age 70+ yrs.	No	No	500,000 IU vitD_3_ vs. placebo once every autumn/winter for 3–5 yrs.	Mental well-being (GHQ, SF-12, WHO Well-Being index.	NS.
Dean et al., 2011 [71]	128 healthy adults, age 18–30 yrs., 57 % female.	No	No	5000 IU/day vitD_3_ vs. placebo for 6 weeks.	Depressive symptoms (BDI).	NS.
Bertone-Johnson et al., 2012 [72]	2263 post-menopausal women, age 50–79 yrs.	No	No	400 IU/day vitD_3_ + 1000 mg/day calcium vs. placebo for 2 yrs.	Depressive symptoms, MDD (Burnam scale, antidepressant use).	NS.
Yalamanchiliet al., 2012 [73]	412 post-menopausal women, age 65–77 yrs.	No	No	Calcitriol 0.25 g twice daily vs. placebo + estrogens vs. placebo for 3 yrs.	Depressive symptoms (GDS).	NS.
Khoraminya et al., 2012 [28]	40 outpatients with MDD, age 18–65 yrs., 85 % female.	No	Yes: MDD diagnosis.	1500 IU/day vitD_3_ + 20 mg fluoxetine vs. fluoxetine alone for 8 weeks.	Depression severity (HDRS), depressive symptoms (BDI).	Vitamin D + fluoxetine superior to fluoxetine alone in reducing depressive symptoms.
Kjærgaard et al., 2012 [29]	230 community dwellers, age 30–75 yrs., 56 % female.	Yes: 25(OH)D <55 nmol/L.	No	40,000 IU/week vitD_3_ vs placebo for 6 months.	Depressive symptoms (BDI, HADS, MADRS).	NS.
Studies published after commencement of the D-Vitaal trial:						
Mozaffari- Khosravi et al., 2013 [30]	109 psychiatric outpatients, age 20–60 yrs., 72 % female.	Yes: 25(OH)D < 40 nmol/L.	Yes: BDI score ≥17.	Single dose of 150,000 or 300,000 IU vitD_3_ vs. no injection.	Depressive symptoms after 3 months (BDI).	Reduced depressive symptoms in 300,000 IU group.

Systematic review of RCTs (inception - July 2015). Search strategy available from author. NS: not significant

PANAS: Positive And Negative Affect Schedule; BDI: Beck Depression Inventory; GHQ: General Health Questionnaire; SF-12: Short Form health survey – 12 item version; MDD: major depressive disorder; GDS: Geriatric Depression Scale; HDRS: Hamilton Depression Rating Scale; HADS: Hospital Anxiety and Depression Scale; MADRS: Montgomery-Åsberg Depression Rating Scale

**Table 2:** Overview of RCTs that examined the effect of vitamin D supplementation on physical functioning

Trial, year	Participants	Low vitD as inclusion criterion?	Poor physical function as inclusion criterion?	Intervention	Outcome	Results
Grady et al., 1991 [74]^a^	98 community dwellers, age 70+ yrs., 54 % female.	No	No	0.25 μg 1,25(OH)_2_D_3_ twice daily vs. placebo for 6 months.	Muscle strength (quadriceps).	NS.
Pfeifer et al., 2000 [75]	148 community dwelling females, age 70+ yrs.	Yes: 25(OH)D <50 nmol/L.	No	800 IU/day vitD_3_ + 1200 mg/day calcium vs. calcium alone for 8 weeks.	Body sway.	Reduced sway in vitD group.
Bischoff et al., 2003 [76]	122 geriatric inpatient females, age 63–99 yrs.	No	No	800 IU/day vitD_3_ + 1200 mg/day calcium vs. calcium alone for 12 weeks.	Musculoskeletal function (knee muscle strength, grip strength, TUG).	Improved musculoskeletal function in vitD group.
Kenny et al., 2003 [77]	60 community dwelling males, age 65–87 yrs.	No	No	1000 IU/day vitD_3_ + 500 mg/day calcium vs. calcium alone for 6 months.	Muscle strength and power (leg, handgrip), physical performance (SPPB, TUG, supine-to-stand test).	NS.
Latham et al., 2003 [78]	243 geriatric in- and outpatients, age 65+ yrs., 53 % female.	No	Yes: frailty.	Single dose of 300,000 IU vitD_2_vs. placebo + exercise training vs. social visits for 10 weeks.	Physical performance (knee strength, balance, TUG, gait speed) after 3 and 6 months.	NS.
Dhesi et al., 2004 [31]	139 outpatients, age 65+ yrs., 78 % female.	Yes: 25(OH)D <30 nmol/L.	Yes: history of falls.	Single intramuscular injection of 600,000 IU vitD_2_ vs. placebo.	Postural sway, physical performance (gait speed, chair stands, stair climbing), quadriceps strength after 6 months.	Improved physical performance and sway in vitD group.
Gallagher, 2004 [79]	289 postmenopausal females, mean age 72 yrs.	No	No	0.5 μg/day calcitriol vs. placebo + estrogen / progesterone treatment vs. placebo for 3 yrs.	Physical performance (grip strength, gait speed, chair stands).	Improved chair stands and gait speed in vitD group (*p* < .1).
Sato et al., 2005 [80]	96 females with post-stroke hemiplegia, mean age 74 yrs.	No	No	1000 IU/day vitD_2_ vs. placebo for 2 yrs.	Muscle strength (of the intact hip).	Improved muscle strength in vitD group.
Bischoff-Ferrari et al., 2006 [81]	64 institutionalized older females, age 65–97 yrs.	No	No	800 IU vitD_3_ + 1200 mg/day calcium vs. calcium alone for 3 months.	Postural and dynamic balance.	NS.
Bunout et al., 2006 [82]	92 community dwellers, age 70+ yrs., 88 % female.	Yes: 25(OH)D <40 nmol/L.	No	400 IU/day vitD_3_ + 800 mg/day calcium vs. calcium alone + exercise training vs. control for 9 months.	Muscle strength (quadriceps, hand grip), gait speed, physical performance (SPPB, TUG), postural sway.	Improved gait speed and sway in vitD group.
Smedshaug et al., 2007 [83]	60 institutionalized persons, mean age 82 yrs., 65 % female.	No	No	400 IU/day vitD_3_ vs. placebo for 1 yr.	Grip strength.	NS.
Brunner et al., 2008 [84]	2364 postmenopausal females, age 50–79 yrs.	No	No	400 IU/day vitD_3_ + 1000 mg/day calcium vs. placebo for 5 yrs.	Grip strength, chair stands, gait speed.	NS.
Moreira-Pfrimer et al., 2009 [85]	46 institutionalized geriatric patients, age 62–94 yrs., 79 % female.	No	No	150,000 IU/month vitD_3_ for 2 months, followed by 90,000 IU/month vs. placebo for 4 months + . 1000 mg/day calcium.	Lower limb muscle strength (hip, knee).	Improved lower limb muscle strength (both hip and knee) in vitD group.
Pfeifer et al., 2009 [86]	242 community dwellers, age 70–94 yrs., 75 % female.	Yes: 25(OH)D <78 nmol/L.	No	800 IU/day vitD_3_ + 1000 mg/day calcium vs calcium alone for 12 months.	Muscle strength (quadriceps), body sway, TUG.	Improved quadriceps strength, sway and TUG performance in vitD group.
Songpatanasilp et al., 2009 [87]^a^	42 postmenopausal females, age 65+ yrs.	Yes: hypovitaminosis D (range NR)	No	0.5 mg/day alfacalcidol + 1500 mg/day calcium vs. calcium alone for 12 weeks.	Muscle strength (quadriceps).	Improved quadriceps muscle strength in vitD group.
Janssen et al., 2010 [88]^a^	70 female geriatric outpatients, age 65+ yrs.	Yes: 25(OH)D 20–50 nmol/L.	No	400 IU/day vitD_3_ + 500 mg/day calcium vs. calcium alone for 6 months.	Muscle strength (knee, leg, hand grip), mobility (TUG), gait speed.	NS.
Lips et al., 2010 [89]	226 community dwellers, age 70+ yrs., % female NR.	Yes: 25(OH)D 15–50 nmol/L.	No	8400 IU/week vitD_3_ vs. placebo for 16 weeks.	Postural sway, SPPB.	Reduced sway in vitD group, only in persons with higher baseline sway.
Witham et al., 2010 [90]	105 patients with systolic heart failure, age 70+ yrs., 34 % female.	Yes: 25(OH)D <50 nmol/L.	No	100,000 IU vitD_2_ vs. placebo at baseline and after 10 weeks.	Gait speed, mobility (TUG), functional limitations.	NS.
Zhu et al., 2010 [91]	261 community dwelling females, age 70–90 yrs.	Yes: 25(OH)D <60 nmol/L.	No	VitD_2_ 1000 IU/day + 1000 mg/day calcium vs. calcium alone for 1 yr.	Lower limb muscle strength (ankle, knee, hip), mobility (TUG).	Improved hip muscle strength and mobility in vitD group, only in persons with lowest baseline function scores.
Glendenning et al., 2012 [92]	686 postmenopausal females, age 70+ yrs.	No	No	150,000 IU vitD_3_ vs. placebo at baseline and after 3 and 6 months.	Grip strength, mobility (TUG).	NS.
Hornikx et al., 2012 [93]	49 COPD patients, age 50+ yrs., 24 % female.	No	No	100,000 IU vitD_3_/month vs. placebo for 3 months.	Muscle strength (inspiratory, quadriceps), gait speed, maximal exercise capacity (cycle ergometer test)	Improved inspiratory muscle strength in vitD group.
Kampman et al., 2012 [94]	68 multiple sclerosis patients, age 21–50 yrs., 71 % female.	No	No	20,000 IU vitD_3_/week vs. placebo for 2 yrs.	Grip strength.	NS.
Studies published after commencement of the D-Vitaal trial:						
Hara et al., 2013 [95]	94 postmenopausal females with osteoporosis, age 55–75 yrs.	No	No	1 μg/day alfacalcidol + 200 mg/day calcium + 35 mg/week alendronate vs. calcium and alendronate alone for 4 months.	Muscle strength (back extensor).	Improved back extensor strength in younger vitD subgroup.
McAlindon et al., 2013 [96]	146 adults with knee osteoarthritis (OA), age 45+ yrs., 61 % female.	No	Yes: presence of symptomatic knee osteoarthritis.	2000 IU/day vitD_3_ with dose escalation to obtain serum levels of >90 nmol/L vs. placebo for 2 yrs.	Gait speed, chair stands.	NS.
Sanghi et al., 2013 [97]	103 adults with knee osteoarthritis, age 40–74 yrs., 64 % female.	No	Yes: presence of symptomatic knee osteoarthritis.	60,000 IU/day vitD_3_ for 10 days followed by 60,000 IU/month vs. placebo for 12 months.	Functional limitations (WOMAC).	Small improvement of physical function in vitD group.
Knutsen et al., 2014 [98]	215 non-western immigrants, age 18–50 yrs., 73 % female.	No	No	1000 or 400 IU vitD3/day vs. placebo for 16 weeks.	Grip strength, chair stands, muscle strength and power.	NS.
Wood et al., 2014 [99]	293 postmenopausal females, age 60–70 yrs.	No	No	400 or 1000 IU/day vitD_3_ vs. placebo for 12 months.	Grip strength.	NS.
Rolighed et al., 2015 [100]	46 patients with primary hyperparathyroidism, age 29–77 yrs., 76 % female.	Yes, 25(OH)D <80 nmol/L.	No	2800 IU/day vitD_3_ vs. placebo for 12 months.	Muscle strength (knee, elbow, hand grip), chair stands, mobility (TUG), postural sway.	NS.

Systematic review of RCTs (inception - July 2015). Search strategy available from author. NS: not significant. NR: not reported.

TUG: Timed Up-and-Go test; SPPB: Short Physical Performance Battery; WOMAC: Western Ontario and McMaster Universities Arthritis Index.

^a^only abstract available

Four out of nine RCTs on depression (44 %) observed a significant positive effect of vitamin D supplementation on depressive symptoms (see Table 1). However, only two studies included persons with either low 25(OH)D or depression at baseline [28, 29], and only one study included persons with both characteristics [30]. The latter study and the study that included persons with a diagnosis of major depressive disorder (MDD) [28] observed significant improvements after the supplementation.

Table 2 shows that in 14 out of 28 RCTs (50 %), vitamin D supplementation improved physical functioning. Twelve studies included participants with either low 25(OH)D or poor physical function at baseline, and physical function improved in seven (58 %) of these studies. Only one study included persons with both low 25(OH)D and poor physical function [31] and this trial observed a significant improvement of physical performance.

In conclusion, the effect of vitamin D supplementation seems more pronounced in persons with low 25(OH)D levels, depressive symptoms and poor physical function at baseline. However, RCTs that specifically included participants with these characteristics are scarce. Furthermore, to the best of our knowledge, no studies have examined the effect of vitamin D supplementation on the combination of mental and physical functioning, despite the fact that these are interrelated concepts.

The current RCT aims to fill this knowledge gap by investigating whether older adults at high risk for poor mental and physical health can benefit from supplementation with vitamin D. Primarily, the D-Vitaal trial examines whether vitamin D supplementation improves depressive symptoms, functional limitations and physical performance in persons with low 25(OH)D levels, clinically relevant depressive symptoms and functional limitations. Other, secondary health outcomes are assessed as well, such as incident MDD [32], anxiety [33], health-related quality of life [34, 35] and cognitive functioning [36]. In addition, it will be investigated whether vitamin D supplementation is a cost-effective strategy for the above-mentioned aims. If proven effective, vitamin D supplementation can easily be implemented in the primary care setting as a simple and safe strategy to prevent both mental and physical disorders in the elderly population.

## Methods/design

### Design of the study

The D-Vitaal study is a randomized, double-blind, placebo-controlled clinical trial that is carried out in the Netherlands. This study examines the effect of a daily dose of 1200 IU vitamin D_3_ versus placebo on depressive symptoms, functional limitations and physical performance. We included 155 participants aged 60–80 years. The duration of the intervention is one year. The D-Vitaal trial was approved by the Medical Ethics Committee of the VU University Medical Centre Amsterdam and is registered with the Netherlands Trial Register under NTR3845. The D-Vitaal study adheres to the CONSORT guidelines for randomized controlled trials.

### Participants

Potential participants were screened for presence of clinically relevant depressive symptoms, functional limitations and low serum 25(OH)D levels (≤50 or ≤70 nmol/L in winter (October-March) and summer (April-September), respectively). In this way, we recruited a vulnerable population at high risk of subsequent mental and physical function decline. As severe 25(OH)D deficiency can be associated with bone disease [37], persons with 25(OH)D levels of <15 nmol/L were excluded and referred to their GP for treatment. In addition, persons with a current MDD diagnosis were excluded, as the aim of this study is to prevent depression. Furthermore, persons who use antidepressant medication were excluded at screening because of possible interference of antidepressants with the effect of vitamin D on depressive symptoms. Persons with sufficient concentrations of 25(OH)D (>50 or >70 nmol/L in winter and summer, respectively) were also excluded, as it was expected that the effect of supplementation in these persons would be negligible. The summer/winter cut-off difference of 20 nmol/L was based on the observation by Van Schoor et al. that the seasonal variation of vitamin D levels in two cohorts of Dutch older persons was 14–24 nmol/L [38]. The Health Council of the Netherlands advises a supplement of 800 IU/day vitamin D for institutionalized persons [39]. Therefore, only community dwelling persons were included in the trial. Table 3 lists the inclusion and exclusion criteria of the D-Vitaal study. All participants provided written informed consent prior to the start of the intervention.

**Table 3:** Inclusion and exclusion criteria of the D-Vitaal study

Inclusion criteria:	Exclusion criteria:
- Men and women of ≥60 and ≤80 years	- Presence of major depressive disorder at screening
- Presence of depressive symptoms (CES-D ≥16)	- Use of antidepressant medication at screening
- Presence of ≥1 functional limitation	- Presence of major life-threatening illness
- Serum 25(OH)D levels ≤50 (winter) or ≤70 (summer) nmol/L.	- Vitamin D (>400 IU/day) or calcium (>1000 mg/day) supplementation
- Ability to comply with the study protocol	- Serum 25(OH)D levels <15 nmol/L.
	- Living in an institution

*CES-D*: Centre of Epidemiological Studies Depression scale

### Recruitment and setting

**Fig. 2:**
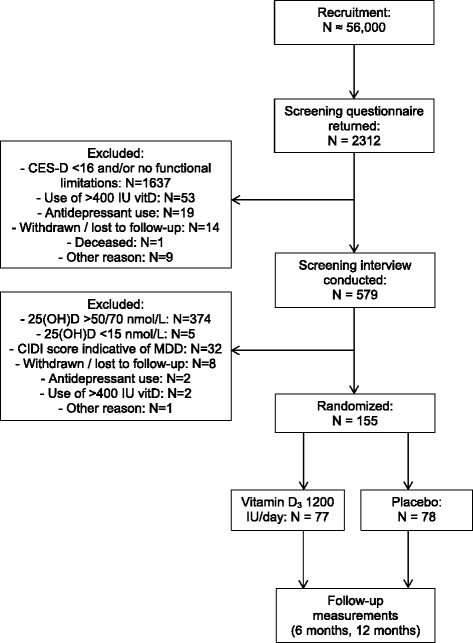
Recruitment, selection and randomization in the D-Vitaal study

The D-Vitaal study is carried out in Amsterdam and surrounding municipalities in the Netherlands. Both urban and rural areas were included. The majority of the participants was recruited through municipality registries. Municipalities in the surroundings of Amsterdam provided the addresses of inhabitants in the age range of 60–80 years. These persons received an information brochure about the study by mail. In addition, advertisement posters and information leaflets were distributed in community centres. About 20 % of participants was recruited through general practitioners (GPs) in Amsterdam. Using a standardized search in their electronic medical records, the GPs selected eligible patients between 60–80 years. These patients received a letter from their GP to draw their attention to the study. Finally, excluded participants of a previous clinical trial [40] who had indicated to be interested in contributing to future research, were invited to participate in the D-Vitaal study. Recruitment commenced in June 2013 and the inclusion was finalized in April 2015. Figure 2 shows a flow chart of the recruitment, selection and randomization in the D-Vitaal study.

### Screening phase

The screening phase of the D-Vitaal study included two steps: a mailed questionnaire and a short interview including a blood sample. The screening questionnaire assessed presence of depressive symptoms (Centre of Epidemiological Studies - Depression scale (CES-D) score of ≥16 [41]) and functional limitations and was also used to exclude persons who use antidepressant medication, vitamin D >400 IU/day and/or calcium supplements >1000 mg/day. For the first 12 participants, we used only the Functional Limitations questionnaire of the Longitudinal Aging Study Amsterdam (LASA-FL) [42] for the assessment of functional limitations. A LASA-FL score of ≥1 indicates the presence of functional limitations. However, this instrument proved not sensitive enough for this study population, creating a large ceiling effect. Especially younger-old participants (e.g. persons in their early sixties) reported few or no problems on this scale, as their level of general functioning was still moderate to high. Therefore, we added the Physical Functioning subscale of the Short Form-36 Health Survey [43] (SF-36-PF) to the screening phase. The SF-36-PF contains 10 items on physical functioning and assesses a broader range of functional limitations. Having difficulty with at least one of the items of the SF-36-PF was regarded as presence of functional limitations. Persons who were eligible according to the screening questionnaire (presence of both depressive symptoms and functional limitations) were invited for a screening visit where presence of MDD was examined and a blood sample was drawn to determine serum 25(OH)D levels.

### Intervention and randomisation

Participants were randomly allocated with a 1:1 ratio to one of two treatment groups: vitamin D_3_ (cholecalciferol) 1200 IU/day or placebo. Participants were stratified by sex and women were stratified by age (60–70 years/71–80 years). An independent pharmacist prepared three randomisation lists with computer-generated numbers using block sizes of four.

The intervention group takes three tablets of 400 IU vitamin D_3_ daily for a duration of 12 months. Based on earlier research, the daily dose of 1200 IU will lead to stable mean 25(OH)D levels of >80 nmol/L in the intervention group, with 90 % of individual 25(OH)D levels above 60 nmol/L within a few months [44, 45]. The placebo group receives identical tablets without vitamin D and it is expected that this group maintains a mean serum 25(OH)D level of about 45 nmol/L. Both the vitamin D and placebo tablets were purchased from Vemedia Manufacturing B.V., The Netherlands. The tablets are supplied in vials of 100 tablets for 6 months, 600 tablets at a time. Participants are allowed to take a (multi)vitamin D supplement with a maximum of 400 IU/day in addition to the study tablets.

#### Co-intervention

Calcium positively influences the bioavailability of vitamin D [46]. In addition, some effects of vitamin D may be caused by increased calcium absorption. Therefore, all participants were advised to use at least three dairy consumptions daily to ensure adequate calcium intake of about 1000 mg/day. Calcium intake was assessed with a structured questionnaire during the screening phase. If calcium intake was low (less than 2 dairy consumptions per day), calcium tablets of 500 mg/day were prescribed to these participants.

### Study procedures

Face-to-face interviews take place at screening, baseline, six and 12 months. The baseline visit was timed closely after the screening phase. Shortly prior to the interviews, participants are asked to complete a mailed questionnaire to reduce interview time. Short telephone interviews are conducted after two weeks, three and nine months to check compliance and adverse events. Table 4 lists all measurements and their time points of the D-Vitaal study. Interviews and blood sampling are carried out by trained researchers or research nurses and take place at the participant’s home, at the VU University Medical Center, or at local community medical centres. The Endocrine Laboratory of the VU University Medical Center conducts the 25(OH)D determinations. Participants who discontinue taking the study tablets for any reason are asked to participate in the remaining follow-up measurements. Any (serious) adverse events are carefully monitored.

**Table 4:** Assessed domains, instruments and their time points in the D-Vitaal study

		*Time point*			
Domain	Instrument	Screening	T0 (baseline)	T1 (6 months)	T2 (12 months)
Primary outcome variables:					
Depressive symptoms	CES-D	X	X	X	X
Functional Limitations	LASA-FL	X	X	X	X
	SF-36 PCS	Partly	X	X	X
Physical performance	SPPB		X	X	X
Secondary outcome variables:					
Major depressive disorder	CIDI	X		X	X
Anxiety	BAI		X	X	X
Health-related quality of life	SF-36		X	X	X
	EQ-5D		X	X	X
Cognition	Stroop test		X	X	X
Physical performance					
Mobility	TUG		X	X	X
Hand grip strength	Dynamometer		X	X	X
Health care costs	TiC-P		X	X	X
Serum 25(OH)D		X		X	
Possible covariables:					
Demographic information					
Age, sex		X			
Education level			X		
Marital status			X	X	X
Lifestyle					
Smoking behaviour			X	X	X
Alcohol consumption			X	X	X
Physical activity	LAPAQ		X	X	X
Blood pressure	Omron device		X	X	X
Anthropometry					
BMI	Height, weight		X	X	X
Waist and calf circumference	Tape measure		X	X	X
Chronic diseases	7 majors		X	X	X
Medication and supplement use			X	X	X
Predictors of vitamin D status			X	X	X
Use of counselling			X	X	X

CES-D: Centre of Epidemiological Studies Depression scale; LASA-FL: Longitudinal Aging Study Amsterdam Functional Limitations questionnaire; SF-36 PCS: Short Form 36 Health Survey - Physical Component Summary score; SPPB: Short Physical Performance Battery; CIDI: Composite International Diagnostic Interview; BAI: Beck Anxiety Inventory; EQ-5D: EuroQol 5 Dimensions; TUG: Timed Up-and-Go test; TiC-P: Trimbos and iMTA questionnaire on Costs associated with Psychiatric illness; LAPAQ: Longitudinal Aging Study Amsterdam Physical Activity Questionnaire

### Compliance

Compliance is checked by tablet count. To stimulate compliance, participants are contacted by telephone (after two weeks, three and nine months) and reminded at follow-up visits. The ultimate compliance check is the measurement of serum 25(OH)D after 6 months.

### Outcomes

#### Primary outcomes

##### Depressive symptoms

The CES-D [41] is used to measure differences in mean change of depressive symptoms after 12 months between the two treatment groups. The CES-D contains 20 items with a score range of 0–60. Higher scores indicate more depressive symptoms. A score of ≥16 is indicative of clinically relevant depressive symptoms. The CES-D is a widely used instrument that displays good psychometric properties in various elderly populations [47].

##### Functional limitations

The LASA-FL questionnaire [42] includes six items assessing common daily activities: climbing stairs, cutting toenails, walking 5 minutes outdoors without resting, rising from a chair, dressing/undressing oneself and using own or public transport. Participants indicate whether they are able to perform these activities without difficulty, with some difficulty, with much difficulty, only with help, or not at all. The instrument can be scored in two ways: by determining the amount of functional limitations (score range 0–6, with higher scores indicating presence of more functional limitations) and the degree of functional limitations (score range 0–24, with higher scores indicating more severe functional limitations). The LASA-FL is used to measure differences in mean change of the amount and degree of functional limitations after 12 months between the two treatment groups.

##### Physical performance

A modified version of the Short Physical Performance Battery (SPPB) [26, 48] is used to determine differences in mean change of objective physical performance between the treatment groups after 12 months. The SPPB includes a walking test (walking 3 meters, turning 180° and walking 3 meters back as fast as possible), a chair stands test (standing up from a chair without using hands five times as fast as possible) and a balance test (standing with feet in tandem stand (i.e. standing with one foot in front of the other, with the heel of one foot touching the toes of the other foot) for up to 30 seconds). Participants can score 0–4 points for each test, adding up to a maximum of 12 points, with higher scores indicating better performance. Reliability and validity of the SPPB are good [49].

#### Secondary outcomes

##### Incidence of MDD

The depression-section of the DSM IV-based Composite International Diagnostic Interview (CIDI, version 2.1) [50] is used to assess presence of MDD. At six and 12 months, the CIDI is administered if a participant scores ≥16 on the CES-D.

##### Anxiety

The Beck Anxiety Index (BAI) [51, 52] is a well-validated questionnaire that contains 21 items measuring anxiety symptoms that are relatively distinguishable from depression symptoms.

##### Cognition

Cognitive function is assessed with indicators of information processing speed and executive functioning obtained from the Stroop-Colour Word Test [53].

##### Health-related quality of life

Health-related quality of life is measured with the EuroQol - 5 Dimensions (EQ-5D) [54] and the SF-36 [43]. The EQ-5D is a utility instrument that enables the calculation of Quality Adjusted Life Years (QALY’s). The SF-36 Mental Component Summary score (MCS) and Physical Component Summary score (PCS) are used as a measure of mental and physical health-related quality of life, respectively [43]. Both instruments are widely used and well-validated in older populations [43, 55].

##### Timed Up-and-Go test

With the Timed Up-And-Go Test (TUG), functional mobility is assessed by asking the participant to stand up from a standard chair, walk a 3 meter distance, turn, walk back to the chair and sit down again [56].

##### Hand grip strength

Hand grip strength is measured in kilograms with a strain-gauged dynamometer (Takei TKK 5401, Takei Scientific Instruments Co. Ltd., Japan). Participants are asked to apply maximum force on the device while in a standing position. Grip strength is measured twice for each hand, consecutively alternating between both hands. The hand grip strength score is derived by taking the mean of the highest score for each hand.

##### Economic evaluation

To evaluate whether vitamin D is a cost-effective intervention for the prevention of depression and poor physical function in older persons, an economic evaluation will be performed. Dutch costing guidelines will be used [57]. All relevant costs will be measured and valued, including the costs of the vitamin D intervention. Health care utilization is measured with the Trimbos and iMTA questionnaire on Costs associated with Psychiatric illness (TiC-P) [58]. Health care costs include costs of GP and psychiatric care, ambulatory and outpatient hospital care, physical therapists and home care. Costs of production loss are not included, as the majority of the participants does not have a paid job.

#### Possible covariables

Several variables are measured during the trial to check for possible (chance-based) differences between the treatment groups. Height and weight are measured with a calibrated stadiometer and scale, respectively. Body mass index (BMI) is calculated by dividing weight (in kilograms) by height^2^ (in meters). Waist and calf circumference is assessed with a tape-measure. Blood pressure and pulse are assessed twice with a three-minute interval using the Omron M1 Plus device (Omron Healthcare Europe). The date of the baseline interview is used to measure seasonal variation of serum 25(OH)D levels [38]. Structured questionnaires assess age, gender, education level, marital status, smoking behaviour, alcohol use, current medication and supplement use, use of corticosteroids in the past three months, chronic diseases, physical activity (walking, cycling, gardening, household activities and sports [59], vitamin D predictors (exposure to sunlight, skin pigmentation, consumption of fatty fish) [60] and use of counselling.

#### Blood sampling and assessment of serum 25(OH)D

Blood samples are obtained in the morning by a trained research nurse at screening and 6 months for measurement of serum 25(OH)D. Participants are in a fasted state with regard to dairy products. Serum 25(OH)D is determined using liquid chromatography followed by tandem mass spectrometry [61]. At screening, 25(OH)D was determined immediately after blood sampling. The 25(OH)D determinations of the 6-month blood samples will be carried out at the end of the study, to ensure randomization concealment. Serum, EDTA plasma and whole blood samples are stored frozen (−80 ° C) until determination and for potential future biomarker and DNA research.

### Power calculation

The primary outcomes of the D-Vitaal study are change in depressive symptoms (CES-D score) and change in physical functioning (functional limitations and physical performance) after 12 months. In an RCT with a comparable population (older persons from the community with depressive symptoms but no MDD), the mean CES-D score was 26 (SD: 5.1) [62]. To detect a change of 0.5 SD (i.e. 2.5 points change of CES-D score), a total of 40 subjects per group is needed, assuming a power of 80 %, a two-sided alpha of .05 and an intraclass correlation coefficient (ICC) of .70 between baseline and follow-up measurements.

The SPPB score ranges from 0 to 12. A change of one point (SD: 1.5) is regarded as a meaningful change [63]. Assuming the same power, alpha and ICC as the CES-D calculation, 22 participants per group are needed.

The LASA-FL questionnaire is scored in two ways: by determining the amount (score range 0–6) and the degree (score range 0–24) of functional limitations. A change of one point in the amount of functional limitations can be regarded as a meaningful change [64]. The SD was set on 1.7, based on analyses in the Longitudinal Aging Study Amsterdam (LASA), a large prospective cohort study of older persons. Assuming again the same power, alpha and ICC as the previous calculations, at least 28 persons per group should be included. For the degree of functional limitations, no meaningful change data or RCTs could be identified in the literature. Based on analyses in LASA, a mean difference of 2 points can be expected, with an SD of 4.5. For this outcome, 48 persons per group are needed, using the same assumptions as indicated above. Considering an expected dropout of 25 % and uncertainty of the 25(OH)D assay, we aimed to include at least 70 participants per group, altogether at least 140 participants.

### Data analysis

Baseline characteristics between treatment groups will be compared using Pearson Chi-square tests, independent-samples t-tests, one-way ANOVAs, or non-parametric tests. Persons who drop out will be compared to persons who complete the study. Skewed data will be transformed. Data will be analysed according to the intention-to-treat principle with longitudinal data analysis techniques (generalized estimating equation (GEE) analysis or mixed model analysis) using SPSS (SPSS Inc. Chicago, IL, USA). A double-sided *p*-value of .05 will be regarded as statistically significant. If needed, models will be adjusted for relevant confounding variables. To investigate the interrelatedness of depressive symptoms and physical functioning, these variables will be added to each other’s effect analysis in separate models. Effect modification will be investigated for age, gender and baseline serum 25(OH)D levels.

Per-protocol analyses will be performed as a secondary analysis with participants who were compliant with the study protocol (≥80 % tablet intake). As a compliance check, the improvement of vitamin D status - i.e. the number of participants with serum 25(OH)D levels over 50, 60 and 75 nmol/L - will also be analysed in secondary analyses. Finally, as a sensitivity analysis, it will be investigated whether change in serum 25(OH)D is associated with change in depressive symptoms, functional limitations and physical performance over time in the total study sample (irrespective of treatment group).

The economic evaluation will be performed from a societal perspective with a time horizon of 12 months. The analysis will be done according to the intention-to-treat principle. Missing cost and effect data will be imputed using multiple imputation according to the MICE algorithm [65]. Bias-corrected and accelerated bootstrapping with 5000 replications will be used to calculate 95 % confidence intervals around the mean difference in total costs between the two groups. Incremental cost-effectiveness ratios (ICERs) will be calculated by dividing the difference in mean total costs by the difference in mean effects on the primary outcomes (depressive symptoms, functional limitations and physical performance) between the treatment groups. A cost-utility analysis will be performed estimating the incremental costs per QALY gained. Bootstrapping will be used to estimate the uncertainty surrounding the ICERs, which will be graphically presented on cost-effectiveness planes. Cost-effectiveness acceptability curves and net monetary benefits will also be calculated. Sensitivity analyses will be performed on the most important and uncertain cost parameters.

## Discussion

As depressive symptoms and poor physical functioning are prevalent among older persons and cause significant individual and societal burden [66], effective low-cost prevention strategies are urgently needed. The D-Vitaal study aims to examine whether vitamin D supplementation decreases depressive symptoms and functional limitations and improves physical performance in older adults. The majority of RCTs that examined vitamin D supplementation included participants with adequate serum 25(OH)D levels and good mental and physical health, which may explain the absence of effects of some previous clinical trials in the field [12].

The D-Vitaal trial is innovative in several ways: it includes persons who may benefit most from the supplementation: older persons with low serum 25(OH)D levels and at high risk for developing poor mental and physical health. Furthermore, the tight interrelationship between depressive symptoms and physical functioning is taken into account by targeting both concepts in one RCT. Physical functioning is measured comprehensively with both self-reported questionnaires and objective tests. Finally, MDD diagnosis is included as a secondary outcome measure. This enables us to explore the effect of the vitamin D supplementation on the development of MDD. To our knowledge, this outcome has not been investigated previously in an RCT [12].

It is more urgent to study the effects of vitamin D supplementation in persons with inadequate vitamin D levels than to examine whether there are any additional effects of supplementation above normal ranges [67]. The amount of supplementation (1200 IU/ day) used in the D-Vitaal study is a moderate dose, but sufficient to correct for deficiency and obtain an adequate vitamin D status [44, 45].

If the results of the D-Vitaal trial indicate that vitamin D supplementation is effective in reducing depressive symptoms and improving physical functioning in older adults, vitamin D can be an efficient intervention that targets two prevalent adverse health conditions simultaneously. The economic evaluation will provide evidence on the cost-effectiveness of the intervention. As vitamin D supplementation is inexpensive and displays minimal side effects, opportunities for implementation in the primary care setting seem promising. The first results of the D-Vitaal study are expected in 2016.

## Abbreviations

1,25(OH)_2_D: 1,25 dihydroxyvitamin D
25(OH)D: 25 hydroxyvitamin D
ANOVA: analysis of variance
BAI: Beck Anxiety Inventory
BMI: body mass index
CES-D: Centre of Epidemiological Studies - Depression scale
CIDI: Composite International Diagnostic Interview
DSM: Diagnostic and Statistical Manual of Mental Disorders
EQ-5D: EuroQol-5 Dimensions
EQ-VAS: EuroQol Visual Analog Scale
GEE: generalized estimating equations
GP: general practitioner
ICC: intraclass correlation coefficient
ICER: incremental cost-effectiveness ratios
LASA-FL: Longitudinal Aging Study Amsterdam Functional Limitations questionnaire
MCS: Mental Component Summary (SF-36)
MDD: major depressive disorder
PCS: Physical Component Summary score (SF-36)
QALY: quality-adjusted life year
RCT: randomized controlled trial
SF-36: Short Form-36 Health Survey
SPPB: Short Physical Performance Battery
TiC-P: Trimbos and iMTA questionnaire on Costs associated with Psychiatric illness
TUG test: Timed Up-and-Go test
VDR: vitamin D receptor
